# The Prognostic Value of Echocardiographic Wall Motion Score Index in ST-Segment Elevation Myocardial Infarction

**DOI:** 10.1155/2022/8343785

**Published:** 2022-11-10

**Authors:** Michael L. Savage, Karen Hay, Bonita Anderson, Gregory Scalia, Darryl Burstow, Dale Murdoch, Isuru Ranasinghe, Owen Christopher Raffel

**Affiliations:** ^1^Cardiology Department, The Prince Charles Hospital, Brisbane, QLD, Australia; ^2^School of Clinical Medicine, Faculty of Medicine, University of Queensland, Brisbane, QLD, Australia; ^3^QIMR Berghofer Medical Research Institute, Brisbane, QLD, Australia

## Abstract

**Background:**

When compared to left ventricular ejection fraction (LVEF), previous studies have suggested the superiority of wall motion score index (WMSI) in predicting cardiac events in patients who have suffered acute myocardial infarction. However, there are limited studies assessing WMSI and mortality in ST-segment elevation myocardial infarction (STEMI). We aimed to compare the prognostic value of WMSI in a cohort of STEMI patients treated with primary percutaneous coronary intervention (PCI).

**Methods:**

A comparison of WMSI, LVEF, and all-cause mortality in STEMI patients treated with primary PCI between January 2008 and December 2020 was performed. The prognostic value of WMSI, LVEF, and traditional risk scores (TIMI, GRACE) were compared using multivariable logistic regression modelling.

**Results:**

Among 1181 patients, 27 died within 30-days (2.3%) and 49 died within 12 months (4.2%). WMSI ≥1.8 was associated with poorer survival at 12-months (9.2% vs 1.5%; *p* < 0.001). When used as the only classifier for predicting 12-month mortality, the discriminatory ability of WMSI (area under the curve (AUC): 0.77; 95% CI: 0.68–0.84) was significantly better than LVEF (AUC: 0.71; 95% CI: 0.61–0.79; *p*=0.034). After multivariable modelling, the AUC was comparable between models with either WMSI (AUC: 0.89; 95% CI: 0.85–0.94) or LVEF (AUC: 0.87; 95% CI: 0.83–0.92; *p* < 0.08) yet performed significantly better than TIMI (AUC: 0.71; 95% CI: 0.62–0.79; *p* < 0.001), or GRACE (AUC: 0.63; 95% CI: 0.54–0.71; *p* < 0.001) risk scores.

**Conclusions:**

When examined individually, WMSI is a superior predictor of 12-month mortality over LVEF in STEMI patients treated with primary PCI. When examined in multivariable predictive models, WMSI and LVEF perform very well at predicting 12-month mortality, especially when compared to existing STEMI risk scores.

## 1. Introduction

The prognostic value of predischarge echocardiography following an acute myocardial infarction (AMI) has been well established [1]. Currently, the European Society of Cardiology guidelines recommend all patients who suffer an ST-segment elevation myocardial infarction (STEMI) should undergo transthoracic echocardiographic evaluation to determine left ventricular ejection fraction (LVEF) prior to discharge [2]. LVEF is widely recognised as a prognostic marker for mortality outcomes following AMI [3–6]. After suffering an AMI, detection of left ventricular regional wall motion abnormalities by echocardiography is common, and wall motion scoring is routinely used to define and quantitate areas of hypokinesia in the affected myocardium [7]. The wall motion score index (WMSI) numerically sums the average scores for all left ventricular segments into a single parameter. The prognostic value of WMSI has been investigated in small cohorts of patients with acute myocardial infarction, suggesting superiority to LVEF in predicting mortality [8–10].

Furthermore, previous findings have indicated that in acute myocardial infarction, WMSI may more accurately reflect the amount of myocardial damage compared to LVEF when compensatory hyperkinesis of noninfarcted myocardium is present [11]. There is also conflicting data whether WMSI is a less sensitive predictor in ST-segment elevation myocardial infarction (STEMI) compared to non-ST segment elevation myocardial infarction (NSTEMI) [8,12]. Despite the suggestion of superiority of WMSI over LVEF to predict mortality, there is limited data pertaining to WMSI in STEMI cohorts treated with primary percutaneous coronary intervention (PCI) and on the routine use of regional wall motion scoring in predictive models postmyocardial infarction. Traditional predictive risk scores used to estimate mortality post-STEMI (GRACE and TIMI Risk Scores [13, 14]) incorporate variables which often fluctuate during initial presentation such as heart rate and systolic blood pressure, but do not include measures of cardiac function such as LVEF or WMSI, which may provide more accurate predictions of survival. We aimed to evaluate the prognostic value of WMSI compared to LVEF in a large cohort of STEMI patients who underwent primary percutaneous coronary intervention (PCI) and compared the performance with traditional STEMI risk scores.

## 2. Methods

### 2.1. Design

A cohort consisting of consecutive STEMI patients treated with primary PCI within 12 hours of symptom onset during a study period between January 2008 and December 2020 at the Prince Charles Hospital, a quaternary referral centre in Queensland, Australia, was analysed. Patients were included for analysis if they survived the index PCI procedure, underwent transthoracic echocardiography within the index admission, and had both LVEF and WMSI calculated on the echocardiogram. Patients were excluded if they did not undergo transthoracic echocardiography during the index admission, did not have either LVEF or WMSI calculated, or were deemed salvage PCI (out-of-hospital cardiac arrest with emergency intubation prior to PCI). This study was conducted according to the World Medical Association Declaration of Helsinki and was approved by the Human Research Ethics Committee of the Prince Charles Hospital (LNR/2018/QPCH/47412) and the Australian Institute of Health and Welfare Ethics Committee (EO2020/2/1147).

### 2.2. Patient Characteristics

Patient demographics, cardiac risk factors, and procedural data were collected at the time of the index admission and recorded in the cardiac catheterisation database. First medical contact was defined as either the time from paramedic arrival for emergency medical service transfer or primary PCI centre arrival for patients who self-presented.

### 2.3. Echocardiographic Measurements

**Figure 1:**
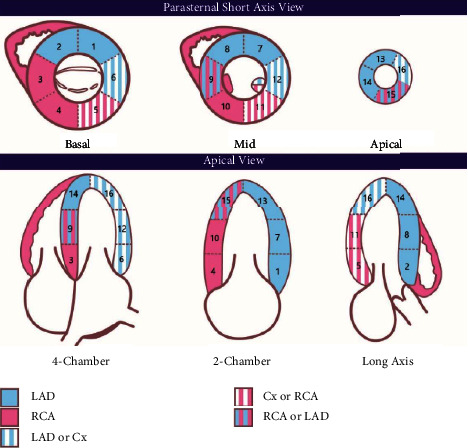
Wall motion scoring segmental analysis by transthoracic echocardiography. LV segments: 1 = basal anterior; 2 = basal anterior septum; 3 = basal inferior septum; 4 = basal inferior; 5 = basal inferolateral; 6 = basal anterolateral; 7 = mid anterior; 8 = mid anterior septum; 9 = mid inferior septum; 10 = mid inferior; 11 = mid inferolateral; 12 = mid anterolateral; 13 = anterior apex (anteroapical); 14 = septal apex (apicoseptal); 15 = inferior apex (inferoapical); 16 = lateral apex (apicolateral). (Modified From Anderson B. Chapter 9 Two-Dimensional Echocardiographic Measurements and Calculations IN *Echocardiography: The Normal Examination and Echocardiographic Measurements (3*^*rd*^*Edition)*: Echotext Pty Ltd; 2017, with permission) [17].

A transthoracic echocardiogram was performed by cardiac sonographers as part of the routine standard of care during the index admission post-PCI. The left ventricular (LV) regional wall motion scores were examined using a 16-segment model as per American Society of Echocardiography (ASE) guidelines (Figure 1) [15, 16]. Individual segments were scored as follows: (1) normal or hyperkinetic, (2) hypokinetic (reduced thickening), (3) akinetic (absent or negligible thickening), and (4) dyskinetic (systolic thinning or stretching). WMSI was calculated by adding the scores of individual segments and dividing this total by the number of segments assessed. LVEF was obtained by Simpson's bi-plane method [17] and is calculated as the percent ratio of the stroke volume (LV end-diastolic volume minus LV end-systolic volume) and LV end-diastolic volume.

### 2.4. Study Outcome

The primary outcome was all-cause mortality. Patients were followed up after primary PCI with the angioplasty nursing service and routine hospital clinic appointments. Outcomes were assessed at 30-days and 12-months post-PCI. Mortality and cause of death data were confirmed with data linkage to the Australian Institute of Health and Welfare's National Death Index registry, which records the death information from all states and territories in Australia.

### 2.5. Statistical Analysis

Patient characteristics and procedural variables were compared by 12-month survival. Continuous variables were summarised as mean (and standard deviation (SD)) and tested between groups using a Student's *t* test if approximately normally distributed, or summarised as median (and interquartile range (IQR)) and tested using Wilcoxon's rank-sum test otherwise. Categorical variables were summarised as frequency (%) and tested between groups using Pearson's chi-square test or Fisher's exact test as appropriate. Where possible, WMSI and LVEF were analysed as continuous variables. Assumptions of linearity were assessed by using the link test and testing fractional polynomial terms to determine the best functional form for continuous variables to be included in logistic regression models. Where cut-points were used, these were chosen based on clinical relevance or prior literature.

Associations between WMSI and LVEF and mortality (30-days and 12 months) were analysed using logistic regression. To facilitate comparison of WMSI and LVEF effect sizes, LVEF was transformed to its complement (by subtracting the value from 100) and both variables were standardised. Confounders of interest were identified *a priori* based on a literature review. These included patient demographics and past medical history, measures of disease severity, and periprocedural variables. Eligible variables with univariable *p* values <0.20 were entered into a multivariable model. Stepwise backward selection was used to determine the base model. Excluded variables were re-entered and tested in the final model which was selected based on Akaike's Information Criteria. Modelling was repeated for each outcome time point (30-day and 12-months) and for each alternative measure of cardiac disease severity (LVEF and WMSI) and compared with existing STEMI risk scores (GRACE and TIMI). The GRACE and TIMI risk scores were chosen as the comparators as they are the most widely validated STEMI risk scores.

Model fit was assessed by comparing the number of observed and predicted events across probability deciles and tested using Hosmer-Lemeshow's goodness of fit test. Internal validation was assessed further by a graphical comparison of calibration belts, which compare predicted and observed values. Parametric receiver operator curve (ROC) analysis was used to compare the discriminatory ability of WMSI to LVEF, both alone and in the presence of covariates, and to compare WMSI to the GRACE and TIMI risk scores. WMSI cut-off values that maximise the sensitivity or specificity were examined according to the Youden or Liu criteria. Nelson-Aaelen methods were used to compare survival over time among groups defined using the optimal cut-points. Analyses were performed using the Stata statistical software package (StataCorp. 2017. *Stata Statistical Software: Release 15*. College Station, TX: StataCorp LLC).

## 3. Results

### 3.1. Study Population

During the study period, 1712 STEMI patients were treated with primary PCI. Of these patients, 1441 (84%) had echocardiographic data available during the inpatient admissions. After exclusions, 1181 patients with both LVEF and WMSI calculated were included in the final cohort. Exclusions are listed in Supplementary Figure 1. Median time from primary PCI to echocardiography was 45 hours (IQR: 22–70). Amongst the 1181 patients, 27 died within 30-days (2.3%) and 49 died within 12 months (4.15%). The distribution of variables of interest by all-cause 12-month mortality is shown in Table 1. Patients who died were on average older had a lower mean GFR.

### 3.2. Echocardiographic Parameters

**Figure 2:**
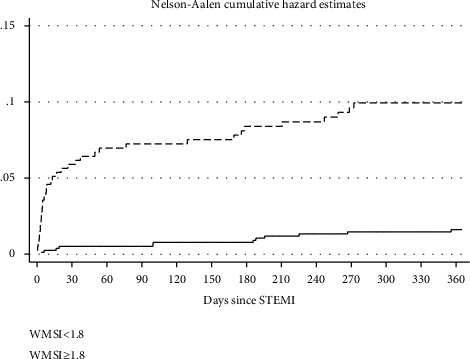
Nelson-Aalen cumulative hazard estimates by subsets indicated demonstrating significantly poorer survival for WMSI ≥1.8 when compared to WMSI <1.8. WMSI–Wall motion score index.

There was a strong inverse correlation between WMSI and LVEF (Pearson's correlation coefficient = −0.78; *p* < 0.001). The mean LVEF was 41% (SD: 14) in those who died compared to 50% (SD: 10) (*p* < 0.001) in those who did not. The median WMSI was 2.2 (IQR: 1.8–2.5) in nonsurvivors compared to 1.6 (1.3–1.9) in survivors at 12-months. WMSI ≥1.8 was associated with increased mortality at 12-months (9.2% vs 1.5%); *p* < 0.001) (Figure 2).

When tested in the same model, LVEF did not remain independently associated with 12-month mortality after adjusting for WMSI. When modelled separately, both WMSI and LVEF were independently and strongly associated with both 30-day and 12-month mortality. After adjusting for age, GFR, and presence of an anterior infarct and cardiogenic shock, the odds of 30-day mortality were 2.4 (95% CI: 1.6–3.6) times higher per 0.4-unit increase in WMSI (Model 1) and 2.1 (95% CI: 1.3–3.2) times higher per 10% decrease in LVEF (Model 2) (Table 2). After adjusting for age, estimated glomerular filtration rate (eGFR), first medical contact (FMC) to balloon time, cardiogenic shock, and smoking status, odds of 12-month mortality were 2.3 (95% CI: 1.7–3.0) times higher per 0.4-unit increase in WMSI (Model 3) and 1.8 (95% CI: 1.3–2.4) times higher per 10% decrease in LVEF (Model 4) (Table 2).

**Figure 3:**
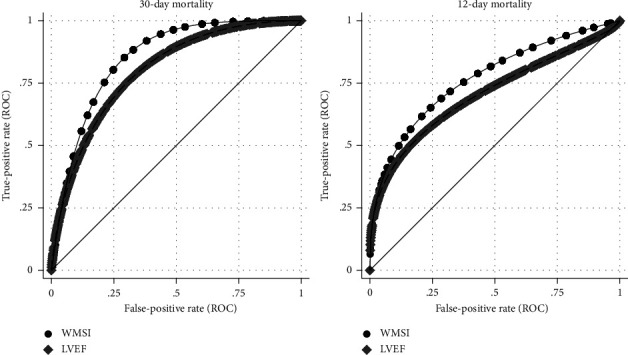
Comparison of receiver operator curves for 30-day and 12-month all-cause mortality by WMSI and LVEF demonstrating higher AUC for WMSI compared to LVEF. WMSI–Wall motion score index, LVEF–Left ventricular ejection fraction.

**Figure 4:**
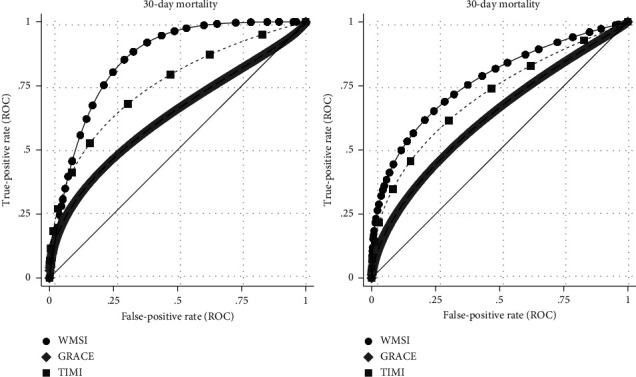
Comparison of receiver operator curves for 30-day and 12-month all-cause mortality by WMSI, GRACE and TIMI risk scores demonstrating higher AUC for WMSI compared to either traditional STEMI risk score. WMSI–Wall motion score index, AUC–Area under curve, STEMI–ST-segment elevation myocardial infarction.

Parametric ROC curves comparing the discriminatory ability of WMSI to LVEF, GRACE, and TIMI scores in correctly classifying patients who died within 30-days and 12-months of their STEMI presentation are shown in Figures 3 and 4. When used as the only classifier for predicting 30-day mortality, the discriminatory ability of WMSI (AUC: 0.85; 95% CI: 0.80–0.91) was significantly higher than the GRACE risk score (AUC:0.63; 95% CI: 0.51–0.76; *p*=0.001) and higher than LVEF (AUC: 0.80; 95% CI: 0.71–0.87) and TIMI (AUC: 0.75; 95% CI:0.65–0.85) but these differences did not reach statistical significance (Table 2). After incorporating other explanatory variables (Models 1 and 2) the AUC indicated excellent and comparable discriminatory ability for models with either WMSI (Model 1—AUC: 0.92; 95% CI: 0.87–0.97) or LVEF (Model 2—AUC: 0.93; 95% CI: 0.88–0.99; *p*=0.23) and was significantly higher than GRACE and TIMI risk scores (Table 2). When used as the only classifier for predicting 12-month mortality, the discriminatory ability of WMSI (AUC: 0.77; 95% CI: 0.68–0.84) was significantly better than LVEF (AUC: 0.71; 95% CI: 0.61–0.79; *p*=0.034) and GRACE (AUC: 0.63; 95% CI: 0.54–0.71; *p*=0.007) but not TIMI (AUC: 0.71; 95% CI: 0.62–0.79). After incorporating other explanatory variables (Models 3 and 4) the AUC was comparable between models with WMSI (Model 3—AUC: 0.89; 95% CI: 0.85–0.94) or LVEF (Model 4—AUC: 0.87; 95% CI: 0.83–0.92; *p*=0.08) (Table 2).

Excellent internal calibration was observed for all multivariable models (Models 1–4) in predicting 12-month mortality, with close agreement between observed proportions and predicted probabilities and confidence intervals symmetrically distributed around the diagonal line.

## 4. Discussion

This study provides an evaluation of the prognostic value of WMSI in comparison to LVEF and traditional STEMI risk models in a large cohort of STEMI patients treated with primary PCI. To our knowledge, this is the largest study examining WMSI, LVEF, and outcomes in the STEMI population. In this study, individually, WMSI, LVEF, and TIMI risk scores were very good predictors of mortality at 30 days post-STEMI and performed significantly better than the GRACE risk score. Consistent with other literature [8,12,18], this study has demonstrated the superiority of WMSI when compared individually to LVEF at predicting 12-month mortality outcomes in STEMI patients. When compared to traditional STEMI risk scores (GRACE and TIMI), the multivariate risk models in this study, adjusting for confounders and mediators by incorporating WMSI and LVEF, performed significantly better (Table 2). The incremental benefit of WMSI over LVEF in predicting mortality when incorporated into a multivariate model, however, was only small. The authors believe that this may be due to inclusion of more relevant confounders such as ischaemic time and renal function, which have previously not been studied when examining WMSI or not traditionally incorporated into the same STEMI mortality risk modelling. The validity of traditional risk scores, in particular the GRACE risk score, may not be as relevant in contemporary STEMI treatment with primary PCI [19], and similar to the findings of this study, displayed weak predictive value of the GRACE score. Other risk scores such as the PAMI and CADILLAC risk scores, although less validated, may have provided a higher predictive value than the traditional risk scores used in our study.

This study also demonstrated a WMSI cut-off of ≥1.8 was a significant and strong predictor of 12-month mortality. Utilising this cut-off may help identify patients who traditionally may have only been identified as having mild LV dysfunction or normal LV function measured by LVEF and may be at increased risk of mortality. The superiority of WMSI compared to LVEF may be explained by several factors, including the nongeometric assumptions with WMSI calculations which are present with LVEF calculations as well as over-compensation of LVEF calculations when incorporating hyperkinetic segments. Additionally, the inferolateral wall is not represented when calculating the LVEF by Simpson's bi-plane method and in patients with isolated inferolateral MI, LVEF will likely be overestimated.

Early studies investigating regional wall motion scoring were positive in demonstrating a correlation with LVEF [20]; however, there were variations in the WMSI calculation models used and were in smaller cohorts of acute myocardial infarction patients. Later studies [21, 22] investigated cohorts of thrombolysed STEMI patients, demonstrating that the use of a 16-segment model to calculate WMSI was better correlated to LVEF than the existing 11- and 14-segment models. Carluccio et al. [22] showed that a WMSI >1.5 was a powerful predictor of subsequent cardiac events which performed better than LVEF <40%. Moller et al. [12] examined a large cohort of 767 acute myocardial infarction patients (376 STEMI) of which 146 patients underwent primary PCI over a median 40-month follow-up. Similar to the results of our study, the authors used a 16-segment model and showed the superiority of WMSI in predicting all-cause mortality (1-year mortality of 18%) over LVEF when incorporated into a Cox regression model.

Several more recent studies have examined WMSI in comparison to other echocardiographic measurements including global longitudinal strain (GLS) and demonstrated good correlation with LVEF, with results of both WMSI and GLS demonstrating superiority to LVEF in predicting combined endpoints of heart failure and mortality in acute myocardial infarction patients with regional wall motion abnormalities [23]. Mistry et al. [24] examined 163 STEMI patients and compared GLS and LVEF and WMSI using a 16-segment model. Whilst GLS best correlated with WMSI (*r*^2^ = 0.55.), WMSI was best correlated with relative infarct size by MRI (*r*^2^ = 0.61.). The authors note that the assumption of geometric symmetry with LVEF calculations may result in a poor correlation with infarct size, whereas WMSI, among other measures, is not geometrically dependent. Similar to the findings of our study, Munk et al. [18] also found WMSI was the strongest single predictor among measures of systolic function (including GLS and LVEF) at predicting cardiac events in a cohort of 526 STEMI patients. More recently, Jurado-Roman et al. [8] examined 278 patients (140 STEMI) with acute myocardial infarction using a 16-segment model and, consistent with the findings of our study, found that a WMSI ≥1.8 on multivariate analysis was the most powerful predictor of mortality and heart failure readmission. Javier Olsen et al. [25] followed 373 STEMI patients treated with primary PCI over a median follow-up of 3.5 years and showed, using a WMSI cut-off of 2.2, a 76% event rate for a combined endpoint of heart failure or cardiovascular death (CVD), although CVD was low at 3.4%. Similar to Munk and colleagues, they demonstrated that when included in multivariable Cox regression models, only WMSI remained an independent significant predictor of outcome (HR = 3.23 (1.56–6.70), *p*=0.002). Our findings support the prognostic value of WMSI demonstrated in previous studies and extend the current literature on the relationship of WMSI with mortality in the context of contemporary treatment of STEMI with primary PCI with comparisons to traditional STEMI risk scores.

We believe, despite the findings of superiority of WMSI over other measures of systolic function including LVEF, issues surrounding the use of WMSI in risk stratification postmyocardial infarction may pertain to the lack of consistency in application of wall motion scoring models and variations in cut-off values in the existing literature. Additionally, when compared to existing WMSI literature, there have been much larger studies examining LVEF and mortality across broader indications than acute myocardial infarction [26, 27]. Similar to our study, other studies [8, 12] have suggested a WMSI cut-off value of ≥1.8 has validity in predicting outcomes at 12-months. A standardised cut-off value for WMSI may provide clinicians with another tool to predict mortality and assist with identifying patients at higher risk than previously identified when stratified by traditional LVEF measurements or using existing STEMI risk scores. The clinical utility of WMSI over other echocardiographic methods of assessment such as strain evaluation is that, despite poor acoustic windows, WMSI can usually be assessed, and it does not require postprocessing using additional software or additional expertise to analyse. In line with the ASE guidelines, we recommend the use of the ASE 16-segment model [15, 16] over the use of the 17-segment model which includes the apex segment, for routine assessment of WMSI scoring, because thickening of the tip of the apex and endocardial excursion are often not well visualized.

## 5. Limitations

This is a single-centre study with a large cohort of consecutive STEMI patients treated with primary PCI. Patient inclusion in the study was reliant on the availability of echocardiographic data at the index presentation. There were patients who were excluded from analysis as WMSI and/or LVEF were either not assessed or not calculated, which may introduce bias. Patients who were intubated prior to primary PCI with unknown neurological status were also excluded to avoid introducing mortality bias (noncardiac related death). Mortality outcomes were lower than previously reported studies examining WMSI in acute myocardial infarction, yet consistent with current primary PCI literature, we presume this was due to the treatment with contemporary primary PCI strategies. This study did not examine confounders such as medication compliance or cardiac rehabilitation, which may also influence mortality post-STEMI. Further investigations incorporating echocardiographic parameters such as WMSI and LVEF into predictive risk models for STEMI are warranted.

## 6. Conclusion

In STEMI patients who underwent primary PCI, both WMSI and LVEF derived from transthoracic echocardiography are highly prognostic for short term mortality. When compared individually, WMSI using the 16-segment model is superior to LVEF measurements at predicting mortality at 12 months. A WMSI cut-off value ≥ 1.8 was correlated with poorer all-cause mortality at 12 months. When compared in multivariable predictive models, the wall motion score index and left ventricular ejection fraction perform very well at predicting 12-month mortality, especially when compared to traditional STEMI risk models.

## Figures and Tables

**Table 1:** Distribution of variables of interest by all-cause 12-month mortality following acute ST-segment elevation myocardial infarction (STEMI) treated with primary percutaneous intervention (PCI).

Variable	Alive (*N* = 1132)	Deceased (*N* = 49)	Total (*N* = 1181)	*p* value
Age^a^ (years)	62.0 (12.2)	74.5 (12.2)	62.5 (12.5)	<0.001
Weight^a^ (kg)	84.4 (17.7)	79.2 (18.8)	84.2 (17.7)	0.041
BMI^b^ (kg/m^2^)	28.3 (5.1)	27.4 (6.0)	28.3 (5.1)	0.20
eGFR^b^ (mL/min/1.73 m^2^)	79.0 (65.0–90.0)	50.0 (43.0–75.0)	78.0 (64.0–90.0)	<0.001
Male sex^c^	881 (77.8%)	34 (69.4%)	915 (77.5%)	0.17
Diabetes^c^	181 (16.0%)	13 (26.5%)	194 (16.4%)	0.051
Hypertension^c^	530 (46.8%)	26 (53.1%)	556 (47.1%)	0.39
Dyslipidemia^c^	496 (43.8%)	21 (42.9%)	517 (43.8%)	0.89
Family history^d^	431 (38.1%)	5 (10.2%)	436 (36.9%)	<0.001
Current smoker^c^	404 (35.7%)	13 (26.5%)	417 (35.3%)	0.19
Previous MI^d^	63 (5.6%)	5 (10.2%)	68 (5.8%)	0.20
Previous CABG^d^	28 (2.5%)	1 (2.0%)	29 (2.5%)	1
Previous PCI^c^	101 (8.9%)	8 (16.3%)	109 (9.2%)	0.08
Multivessel disease^c^	376 (33.2%)	25 (51.0%)	401 (34.0%)	0.01
Multivessel PCI^c^	38 (3.4%)	3 (6.1%)	49 (4.1%)	0.30
TIMI III post PCI	1075 (95.0%)	42 (85.7%)	1117 (94.6%)	0.005
Cardiogenic shock^c^	13 (1.1%)	6 (12.2%)	19 (1.6%)	<0.001
DTB^b^ (minutes)	46 (32–73)	66 (42–83)	47 (32–74)	0.002
FMCTB^b^ (minutes)	100 (84–123)	138 (107–167)	101 (85–125)	<0.001
STB^b^ (minutes)	172 (131–262)	220 (164–271)	173 (132–266)	0.012
Anterior infarct^c^	470 (41.5%)	30 (61.2%)	500 (42.3%)	0.006
Drug eluting stent^c^	818 (72.3%)	29 (59.2%)	847 (71.7%)	0.047
Atrial fibrillation^c^	56 (4.9%)	5 (10.2%)	61 (5.2%)	0.1
LVEF%^a^	50.3 (10.2)	40.9 (13.7)	49.9 (10.5)	<0.001
WMSI^b^	1.6 (1.3–1.9)	2.2 (1.8–2.5)	1.6 (1.3–1.9)	<0.001
GRACE^b^	127 (110–144)	139 (118–165)	127 (110–144)	0.003
TIMI^b^	3.0 (2.0–5.0)	6.0 (4.0–8.0)	3.0 (2.0–5.0)	<0.001
WMSI ≥1.8^c^	364 (32.2%)	37 (75.5%)	401 (34%)	<0.001
LVEF <40%^c^	166 (14.7%)	25 (51.0%)	191 (16.2%)	<0.001

Summary statistics: ^a^mean (SD) with *p* value derived from Student's *t*-test; ^b^median (IQR) with *p* value derived from Wilcoxon's rank-sum test; frequency (%) with *p* values from ^c^Pearson's chi-square test or ^d^Fisher's exact test. BMI–Body Mass Index; eGFR–Estimated Glomerular Filtration Rate; MI–Myocardial Infarction; CABG–Coronary artery bypass grafting; PCI–Percutaneous Coronary Intervention; DTB–Door to balloon; FMCTB–First Medical Contact to Balloon; STB–Symptom to Balloon; LVEF–Left Ventricular Ejection Fraction; WMSI–Wall Motion Score Index.

**Table 2:** Univariable and multivariable associations with all-cause 30-day and 12-month mortality following acute ST-segment elevation myocardial infarction (STEMI) treated with primary percutaneous intervention (PCI) (*N* = 1181).

Variable	Univariable			*p* value^e^	Multivariable			*p* value^e^
	OR (95% CI)	*p* value	AUC (95% CI)		OR (95% CI)	*p* value^e^	AUC (95% CI)	
*30 Day mortality*								
WMSI^a^	2.8 (2.1–3.9)	<0.001	0.85 (0.80–0.91)		2.4 (1.6–3.6)	<0.001	Model 1:	
Age^b^	2.6 (1.8–3.7)	<0.001			1.9 (1.2–3.1)	0.004	0.92 (0.87–0.97)	
Anterior infarction	6.2 (2.3–16.5)	<0.001			3.6 (1.2–10.7)	0.021		
eGFR^c^	1.9 (1.6–2.4)	<0.001			1.5 (1.2–1.9)	0.001		
Cardiogenic shock	18.5 (6.1–55.9)	<0.001			5.4 (1.1–26.7)	0.039		

LVEF^d^	3.0 (2.1–4.4)	<0.001	0.80 (0.71–0.87)	0.085	2.1 (1.3–3.2)	0.001	Model 2:	0.23
Age^b^					1.8 (1.2–2.7)	0.007	0.93 (0.88–0.99)	
Anterior infarction					3.9 (1.3–11.5)	0.013		
eGFR^c^					1.5 (1.2–2)	<0.001		
Cardiogenic shock					5.5 (1.2–24.9)	0.027		

GRACE risk score			0.63 (0.51–0.76)	0.001			0.63 (0.51–0.76)	<0.001
TIMI risk score			0.75 (0.65–0.85)	0.09			0.75 (0.65–0.85)	0.002

*12-Month mortality*								
WMS^Ia^	2.4 (1.9–3.1)	<0.001	0.77 (0.68–0.84)		2.3 (1.7–3.0)	<0.001	Model 3:	
Age^a^	2.4 (1.9–3.1)	<0.001			2.1 (1.5–3.0)	<0.001	0.89 (0.85–0.94)	
FMCTB>120 mins	4.6 (2.5–8.3)	0.08			3.7 (1.9–7.3)	<0.001		
eGFR^c^	1.7 (1.5–1.9)	<0.001			1.3 (1.1–1.5)	0.004		
Cardiogenic shock	12.0 (4.4–33.1)	<0.001			4.3 (1.2–15.6)	0.025		
Current smoker	0.7 (0.3–1.2)	0.19			2.2 (1–5.1)	0.061		

LVEF^d^	2.4 (1.7–2.9)	<0.001	0.71^e^ (0.61–0.79)	0.034	1.8 (1.3–2.4)	<0.001	Model 4:	0.08
Age^a^					2.0 (1.4–2.8)	<0.001	0.87 (0.83–0.92)	
FMCTB>120 mins					3.3 (1.7–6.2)	<0.001		
eGFR^c^					1.3 (1.1–1.5)	0.001		
Cardiogenic shock					5.0 (1.5–17.1)	0.01		
Current smoker					2.1 (0.9–4.9)	0.07		

GRACE risk score			0.63 (0.54–0.71)	0.007			0.63 (0.54–0.71)	<0.001
TIMI risk score			0.71 (0.62–0.79)	0.28			0.71 (0.62–0.79)	<0.001

Effects of continuous variables are reported as ^a^per 0.4-unit increase; ^b^per 10-year increase; ^c^per 10 mL/min/1.73 m^2^ unit decrease; ^d^per 10% decrease. WMSI–Wall Motion Score Index; FMCTB–First Medical Contact to Balloon (minutes); eGFR–Estimated Glomerular Filtration Rate; ^e^*p* values for comparison of ROC areas for classifiers (LVEF, GRACE, TIMI) compared to WMSI (obtained from parametric ROC regression).

## Data Availability

The clinical data used to support the findings of this study are restricted by the Prince Charles Hospital Human Research and Ethics Committee in order to protect patient privacy and confidentiality. Data are available from the relevant Prince Charles data custodian/s for researchers who meet the criteria for access to confidential data.

## Supplementary Materials

Supplementary Materials Supplementary Figure 1. Reasons for exclusion from cohort. PPCI–Primary percutaneous coronary intervention, STEMI–ST-segment elevation myocardial infarction, LVEF–Left ventricular ejection fraction, and WMSI–Wall motion score index.
