# Supplementary figures and images for: The Prognostic Value of Echocardiographic Wall Motion Score Index in ST-Segment Elevation Myocardial Infarction
Source: Crit Care Res Pract. 2022 Nov 10;2022:8343785. doi: 10.1155/2022/8343785 (PMC9671736; doi:10.1155/2022/8343785)

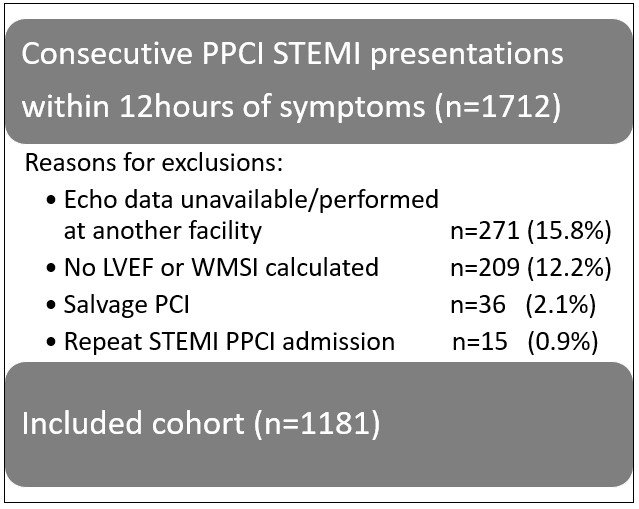

Supplement: Supplementary Materials — Supplementary Figure 1. Reasons for exclusion from cohort. PPCI–Primary percutaneous coronary intervention, STEMI–ST-segment elevation myocardial infarction, LVEF–Left ventricular ejection fraction, and WMSI–Wall motion score index. [file 8343785.f1.jpg]
